# Structural and functional dissection of the WH2/DAD motif of INF2, a formin linked to human inherited degenerative disorders

**DOI:** 10.1111/febs.70271

**Published:** 2025-09-24

**Authors:** Leticia Labat‐de‐Hoz, Laura Fernández‐Martín, Paula Morales, Isabel Correas, María Ángeles Jiménez, Miguel Angel Alonso

**Affiliations:** ^1^ Centro de Biología Molecular Severo Ochoa (CBMSO) Consejo Superior de Investigaciones Científicas (CSIC) and Universidad Autónoma de Madrid (UAM) Spain; ^2^ Instituto de Química Médica (IQM) CSIC Madrid Spain; ^3^ Department of Molecular Biology UAM Madrid Spain; ^4^ Instituto de Química Física (IQF) Blas Cabrera CSIC Madrid Spain

**Keywords:** actin, diaphanous autoregulatory domain, formins, genetic variants, micronucleus, NMR, renal disease, WH2 domain

## Abstract

Inverted formin‐2 (INF2), a formin linked to inherited renal and neurological disorders, exhibits pathogenic variants that lead to deregulated actin polymerization and nuclear aberrations, ultimately compromising cell viability. Most formins contain a diaphanous autoregulatory domain (DAD) and a diaphanous inhibitory domain (DID), which interact to keep the molecule in an inactive state. The DAD consists of a short sequence with an N‐terminal region rich in hydrophobic residues and a C‐terminal segment abundant in basic residues, resembling WASP homology 2 (WH2) actin‐binding domains. Based on its sequence and actin‐binding ability, the DAD of INF2 qualifies as a WH2 motif. In this study, we investigated the structure of the INF2 WH2/DAD by nuclear magnetic resonance (NMR) and explored its functional role. Our analysis revealed that the WH2/DAD forms a single α‐helix in both H_2_O and 30% 2,2,2‐trifluoroethanol that differs from the conformations observed in WH2–actin and DAD–DID crystal structures. Cells expressing INF2 containing only the hydrophobic region of the WH2/DAD exhibited higher F‐actin levels and frequencies of nuclear abnormalities, phenocopying the effect of pathogenic INF2 DID variants. In contrast, deletion of the entire WH2/DAD, or of the hydrophobic region alone, abolishes INF2 activity. Neither these deletions nor WH2/DAD variants carrying naturally occurring missense mutations induced any detectable nuclear effects. These findings suggest that the WH2/DAD undergoes a conformational change to facilitate actin binding and that the hydrophobic region is essential for INF2‐mediated actin polymerization. INF2 WH2/DAD variants with deleterious cellular effects appear to be rare in, or absent from, the human population.

## Graphical abstract

The INF2 WH2/DAD domain, composed of hydrophobic and basic regions, forms a single α‐helix in solution. The hydrophobic segment is essential for INF2 function, as its deletion disrupts actin dynamics and causes nuclear abnormalities, like those seen in pathogenic INF2 DID variants. In contrast, natural INF2 WH2/DAD variants found in patients with renal and neurological disorders appear not to induce such nuclear defects, suggesting a limited effect in cells.

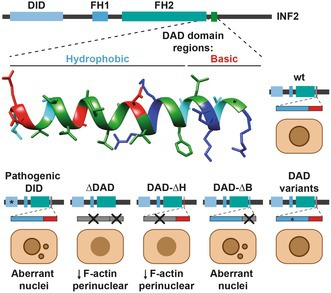

**Abbreviations**
CaM: calmodulin
CaMBS: calmodulin‐binding site
CD: circular dichroism
DAD: diaphanous autoregulatory domain
DAPI: 4′,6‐diamidino‐2‐phenylindole dihydrochloride
DID: diaphanous inhibitory domain
FH: formin homology
GFP: green fluorescence protein
GST: glutathione S‐transferase
INF2: inverted formin‐2
KO: knockout
NMR: nuclear magnetic resonance
NOE: nuclear Overhauser effect
NOESY: nuclear Overhauser effect spectroscopy
TFE: 2,2,2‐trifluoroethanol
WH2: WASP homology 2

## Introduction

Formins are an evolutionarily conserved family of proteins that catalyze the assembly of linear actin filaments. They play critical roles in key cellular processes such as mitosis, cytokinesis, cell adhesion, migration, cell polarity maintenance, membrane trafficking, and embryonic development [1]. Due to their essential functions, mutations in formin genes are linked to a variety of inherited human disorders, as well as being associated with developmental defects, age‐related diseases, and cancer [2, 3]. Of the formins linked to monogenic diseases, INF2 is of particular note because it is implicated in two inherited degenerative disorders: focal segmental glomerulosclerosis, which impairs kidney function, and Charcot–Marie–Tooth disease, which affects the peripheral nervous system [4, 5, 6]. In cultured cells, the expression of pathogenic INF2 variants leads to deregulated actin polymerization activity, altering the transcriptome, and disrupting mitosis, mitochondrial homeostasis, and specialized membrane trafficking pathways, ultimately causing cell death [7, 8].

The core region of formins is made up of formin‐homology 1 (FH1) and formin‐homology 2 (FH2) domains, which comprise the minimal unit required for actin polymerization. Flanking this core, many formins—including INF2—feature a short diaphanous autoregulatory domain (DAD) at the C‐terminal end and a diaphanous inhibitory domain (DID) at the N‐terminal end [1, 9]. In the formin mDia1, the DAD interacts with the DID to establish an autoinhibited conformation [10, 11, 12]. However, in INF2, the DID–DAD interaction is significantly weaker, and the inactive state requires additional regulatory proteins for stabilization [13]. Beyond its role in autoinhibition, the DAD also contributes to actin nucleation, coordinating with the profilin‐bound FH1 domain and the catalytic FH2 domain to form a tripartite nucleation apparatus [14].

WASP homology 2 (WH2) domains are short motifs that interact with monomeric actin and are found in various eukaryotic actin nucleators, such as Spire, Cobl, Lmod, WASP, WAVE, as well as in the VopL/VopF and Sca2 bacterial nucleators [15, 16]. DAD and WH2 domains have sequence similarities, both being organized into an N‐terminal hydrophobic‐rich region and a C‐terminal region with basic residues [9, 16]. Notably, the INF2 DAD contains a canonical WH2 domain, which gives INF2 the apparently unique ability among formins to sever actin filaments *in vitro* [17, 18, 19]. Despite significant progress toward understanding INF2 function, our knowledge of the precise structure of the INF2 WH2/DAD and its regulatory role in actin regulation remains incomplete.

In this study, we investigated the structure of the isolated INF2 WH2/DAD peptide and examined its functional significance by expressing deletion mutants of INF2 lacking either the entire WH2/DAD or its N‐terminal or C‐terminal regions. We also assessed whether the expression of natural INF2 WH2/DAD variants has deleterious effects on cultured cells similar to those of pathogenic DID variants. Our findings revealed that the entire WH2/DAD domain adopts a single α‐helix structure in both H_2_O and 30% 2,2,2‐trifluoroethanol (TFE) and that while both the N‐ and C‐terminal regions are both necessary for actin binding, only the N‐terminal region is critical for INF2‐mediated actin polymerization. None of the natural INF2 WH2/DAD mutants tested exhibited deleterious effects in cultured cells, despite being selected for their potential pathogenicity. These results suggest that the WH2/DAD of INF2 probably undergoes a conformational change upon actin binding and that the presence of the N‐terminal region of the WH2/DAD is essential for INF2‐mediated actin polymerization.

## Results

### The WH2/DAD of INF2 is conserved in vertebrates

**Fig. 1:**
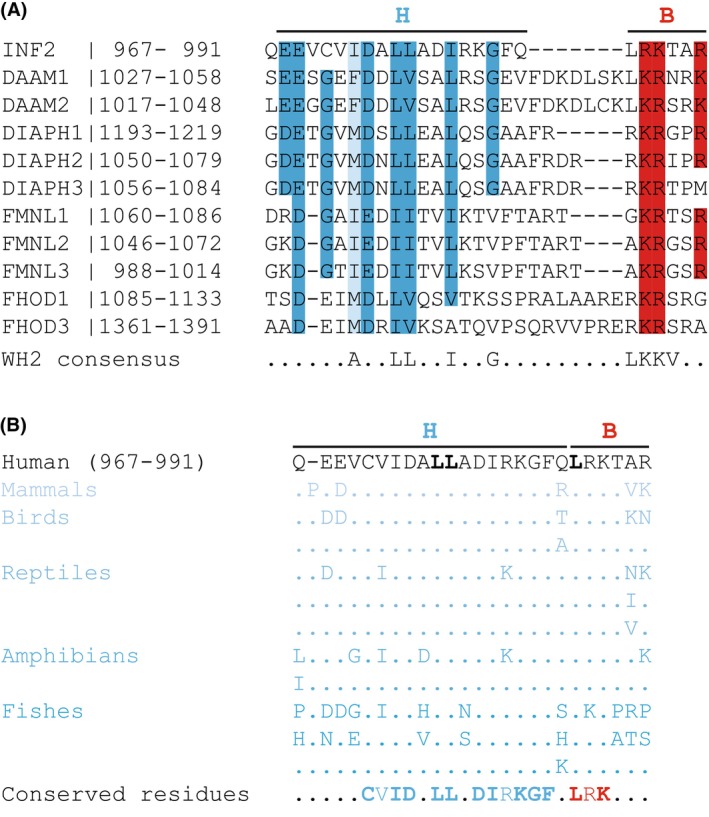
Comparison of the WH2/DAD of human INF2 with the DAD of other human formins and its conservation across vertebrates. (A) Sequence alignment of the human INF2 WH2/DAD (residues 967–991) with the DAD sequences of the other DAD‐containing human formins. Uniprot accession numbers: INF2 (Q27J81), DAAM1 (Q9Y4D1), DAAM2 (Q86T65), DIAPH1 (O60610), DIAPH2 (O60879), DIAPH3 (Q9NSV4), FMNL1 (O95466), FMNL2 (Q96PY5), FMNL3 (Q8IVF7), FHOD1 (Q9Y613), FHOD3 (Q2V2M9). Dark blue shading (hydrophobic region, H) and red shading (basic region, B) highlight identical residues or with very strictly conserved substitutions (e.g., E and D, R, and K, or L, I and V). Light blue shading indicates less strict amino acid hydrophobic substitutions (e.g., I, M, and F). Only residues conserved in at least six of the eleven DAD‐containing human formins are highlighted. The bottom line shows amino acids conserved in more than 50% of 100 representative WH2 domain sequences from different proteins and species [16]. (B) Summary of the amino acid substitutions in the human INF2 WH2/DAD sequence across 45 vertebrate species, including 19 mammals, 9 birds, 7 reptiles, 4 amphibians, and 5 fish species (see Fig. S1 for detailed alignments). The leucine residues (Leu976, Leu977, and Leu986) of INF2 critical for WH2 function are indicated in bold. The bottom line summarizes the conservation of the human INF2 WH2/DAD residues across vertebrates. Identical residues (bold letters) or very strictly conserved substitutions (normal letters) are highlighted in blue (H region) and red (B region).

Most formins contain a DAD sequence in their C‐terminal region [1, 9]. For instance, INF2 and ten more of the fifteen human formins possess a DAD [20]. This domain consists of an N‐terminal region (referred to here as the hydrophobic or H region) that is rich in aliphatic hydrophobic amino acids, primarily leucine residues arranged in a LLXXL motif, and a C‐terminal stretch enriched in basic amino acids (referred to here as the basic or B region). While the B region lacks a clearly defined consensus sequence, it consistently includes a basic dipeptide (Fig. 1A). The structural organization of the DAD into H and B regions mirrors that of WH2 domains, which also feature an N‐terminal LLXXL box and a basic C‐terminal B region (Fig. 1A). The B region of WH2 domains contains a tetrapeptide with an invariant leucine residue followed by a basic dipeptide and typically ends in valine or threonine, though occasionally with other residues (LKKV motif) [16]. The DADs of INF2, DAAM‐1, and DAAM2 align with the consensus sequence of WH2 domains (Fig. 1A). The DAD of INF2, which contains the LLADI and LRKT sequences corresponding to the LXXL and LKKV boxes, respectively, is known to harbor an actin‐binding WH2 domain [17]. In contrast, the DADs of other human formins exhibit only WH2‐like sequences. Phylogenetic analysis of the WH2/DAD (residues 967–991) of human INF2 across vertebrates reveals a high degree of conservation, particularly in three leucine residues known to be critical for WH2 domain function—the leucine dipeptide (Leu976‐Leu977) of the LLXXL box and the leucine residue (Leu986) within the LKKV box [21]—as well as in the basic dipeptide (Arg987‐Lys988) of the LKKV box (Fig. 1B and Fig. S1).

### The WH2/DAD of INF2 adopts a single α‐helix structure in H_2_O and 30% TFE

**Fig. 2:**
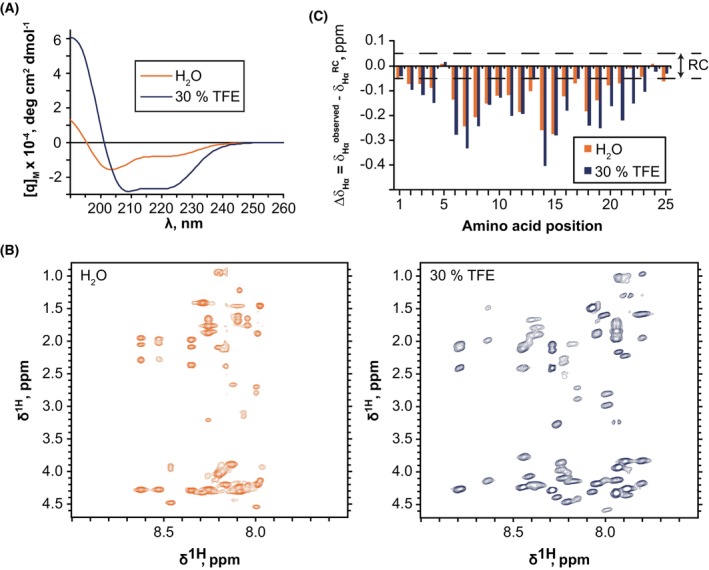
Circular dichroism and nuclear magnetic resonance (NMR) analysis of the INF2 WH2/DAD peptide. (A) Circular dichroism spectra of the INF2 WH2/DAD peptide in aqueous solution (orange line) and in the 70:30 mixed solvent TFE/H_2_O (blue line) at pH 5.5 and 25 °C. Four scans were performed. (B) 2D ^1^H,^1^H‐ total correlation spectroscopy spectra acquired for the INF2 WH2/DAD peptide in aqueous solution (orange contours) and in 30% TFE (blue contours) at pH 5.5 and 25 °C. (C) Bar plots showing the Δ*δ*_Hα_ values as a function of the amino acid position in aqueous solution (orange bars) and in 30% TFE (blue bars) at pH 5.5 and 25 °C. (*n* = 1; averaged error in chemical shift measurements is ±0.01 ppm). Residues 1–25 of the peptide correspond to residues 967–991 of INF2. ppm, parts per million; RC, random coil value interval; TFE, 2,2,2‐trifluoroethanol. [Correction added on 2 October 2025 after first online publication: Panel 2A has been corrected.]

To investigate the structure of the human INF2 WH2/DAD in solution, we used the 25‐residue 967–991 peptide, incorporating a Cys971Ser substitution to prevent artifacts from oxidation or disulfide bond formation. The circular dichroism (CD) spectrum of the WH2/DAD peptide in aqueous solution indicated that the peptide was mainly disordered with a low‐populated α‐helix (Fig. 2A), consistent with the predictions of the AGADIR algorithm (Table 1). In 30% TFE, which mimics hydrophobic environments and promotes α‐helical conformations if peptides have a tendency to form them [22], the CD spectrum exhibited the characteristic features of α‐helices, including two minima at 208 and 222 nm, and a maximum near 195 nm (Fig. 2A).

**Table 1:** Predicted and experimental helix percentages for the INF2 WH2/DAD peptide in H_2_O and 30% TFE at pH 5.5 and 25 °C. CD, circular dichroism; NMR, nuclear magnetic resonance; ppm, parts per million; TFE, 2,2,2‐trifluoroethanol; WH2/DAD, WASP homology 2/diaphanous autoregulatory domain.

		WH2/DAD	
		H_2_O	30% TFE
AGADIR prediction	% helix (residues 1–25)	20	
	% helix (residues 6–17)	39	
CD	[*θ*]^222nm^, deg cm^2^·dmol^−1^	−8 × 10^3^	−26.5 × 10^3^
	% helix (residues 1–25)	24	67
NMR	Helix length^a^	3–24	3–24
	Averaged Δ*δ*_Hα_, ppm	−0.14	−0.21
	% helix	36	53

^a^ Residues 1–25 of the peptide correspond to residues 967–991 of INF2.

**Fig. 3:**
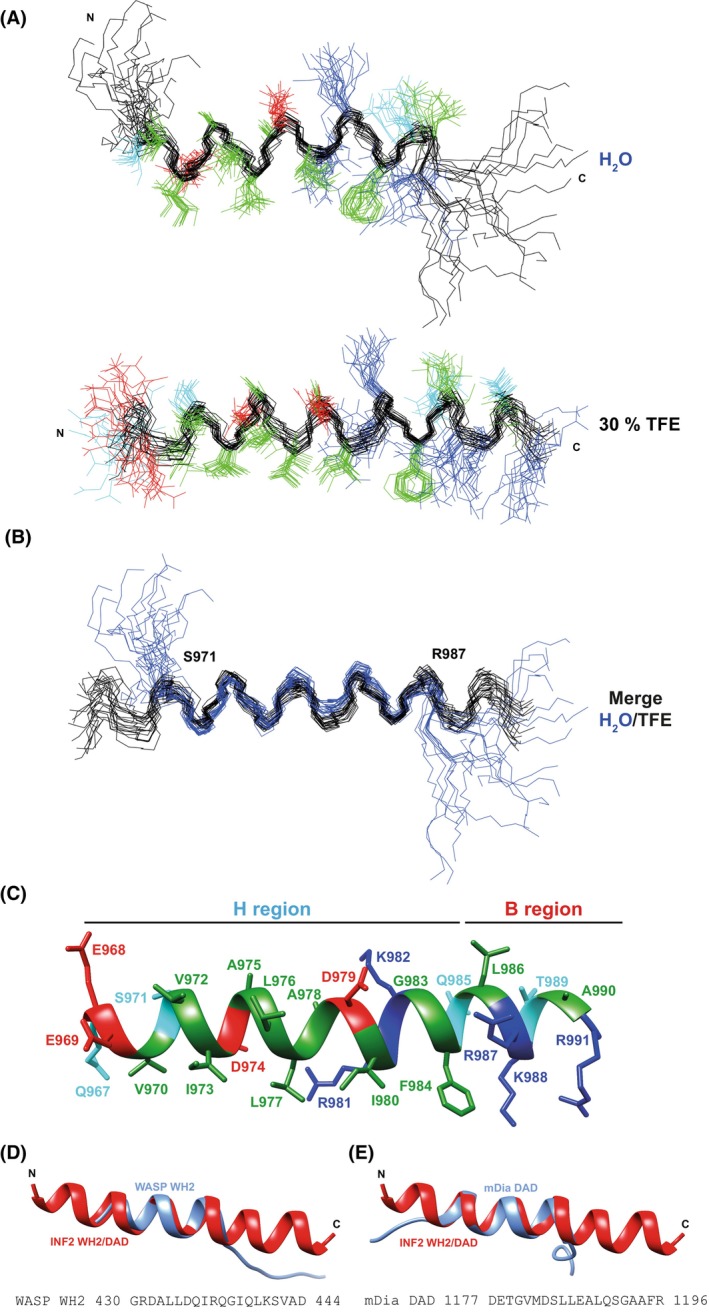
Structure of the INF2 WH2/DAD peptide revealed by NMR. (A) Overlay of the backbone atoms (black) from the 20 lowest target function conformers of the structure in aqueous solution (top panel) and in 30% TFE (bottom panel). (B) Overlay of the backbone atoms of the ensembles in aqueous solution (blue) and 30% TFE (black). Conformers in (A, B) were visualized with MOLMOL. (C) Ribbon representation of the first conformer of the ensemble obtained in 30% TFE. (D, E) Superimposition of the NMR structure of the INF2 WH2/DAD on the crystallographic structure of the WASP WH2 domain (PDB 2VCP) when bound to G‐Actin (D) and the mDia1 DAD (PDB 2F31) when bound to the diaphanous inhibitory domain (DID) of mDia1 (E). In (A) and (C), hydrophobic sidechains are shown in green, polar sidechains in cyan, positively charged sidechains in blue, and negatively charged sidechains in red. Statistical data for the structural ensembles in (A) and (B) are presented in Table 2. The lowest target function conformer is shown in (C–E) using chimerax. The hydrophobic (H) and basic (B) regions are indicated. DAD, diaphanous autoregulatory domain; TFE, 2,2,2‐trifluoroethanol; WH2, WASP homology 2.

To analyze the structure of the WH2/DAD peptide further, we investigated the WH2/DAD peptide by NMR in aqueous solution and in 30% TFE. After assigning the ^1^H shifts (Fig. 2B), secondary structure elements were identified from the Δ*δ*_Hα_ values, which represent deviations in chemical shifts from random coil reference values (Fig. 2C). The negative Δ*δ*_Hα_ values indicated that the INF2 WH2/DAD peptide forms an α‐helical structure both in aqueous solution and in 30% TFE (Fig. 2C). The magnitude of the Δ*δ*_Hα_ values suggests an increase in helix population in the presence of TFE, as confirmed by the estimated α‐helix percentages (Table 1), which align with those derived from CD spectra. The 3D structure of the WH2/DAD peptide was determined in aqueous solution and in 30% TFE based on distance restraints from NOE signals in NOESY spectra and dihedral angle restraints derived from ^1^H and ^13^C chemical shifts (Table 2). In aqueous solution, the WH2/DAD peptide forms a well‐defined amphipathic α‐helix that spanned residues 6–21 of the peptide (residues 972–987 of INF2), with the five‐amino acid N‐ and C‐terminal segments being mainly disordered (Fig. 3A, top panel). In the presence of 30% TFE, the helix is longer (Fig. 3A, bottom panel), spanning residues 3–24 of the peptide (residues 969–990 of INF2). The α‐helical region comprising residues 6–21 of the peptide is essentially identical in both aqueous solution and 30% TFE (Fig. 3B). When we superimposed the structure in 30% TFE (Fig. 3C) onto the crystallographic structure of the WASP WH2 bound to actin (Fig. 3D) and that of mDia1 DAD bound to the mDia DID (Fig. 3E), we observed complete and partial overlap in the H regions, respectively. In crystal structures of the WASP WH2 domain bound to actin and mDia1 DAD bound to the DID, a bend is observed between the H and the B regions. In both cases, the H region adopts an α‐helical structure, while the B region remains extended [10, 11, 12, 23, 24, 25, 26]. By contrast, the INF2 WH2/DAD forms a continuous α‐helix in solution—presumably reflecting its unbound conformation—suggesting that the WH2 region of WASP and the DAD of mDia1 might likewise adopt α‐helical conformations in their unbound states. However, confirming this would require determining the structures of these peptides under conditions comparable to those used for INF2 WH2/DAD.

**Table 2:** Summary of structural statistics of the ensemble of the 20 lowest target function conformers calculated from the INF2 WH2/DAD peptide in H_2_O and 30% TFE at pH 5.5 and 25 °C. RMSD, root mean square deviation; TFE, 2,2,2‐trifluoroethanol; WH2/DAD, WASP homology 2/diaphanous autoregulatory domain.

		H_2_O (PBD 9RWF)	30% TFE (PBD 9G7T)
Number of distance restraints	Intra‐residue (i – j = 1)	65	110
	Sequential (i – j < 1)	52	63
	Medium range (1 < |i − j| < 5)	18	62
	Long range (|i – j| ≥ 5)	0	0
	Total number	135	235
	Averaged total number per residue	5.4	9.4
Number of dihedral angle constraints	Number of restricted phi angles	23	23
	Number of restricted psi angles	18	23
	Total number	41	46
Pairwise RMSD (Å)	Helical residues^a^	5–21	3–24
	Backbone atoms	1.0 ± 0.4	1.0 ± 0.4
	All heavy atoms	1.9 ± 0.4	1.8 ± 0.3
Ramachandran plot (%)	Most favored regions	90.0	100
	Additionally allowed regions	10.0	0.0
	Generously allowed regions	0.0	0.0
	Disallowed regions	0.0	0.0

^a^ Residues 1–25 of the peptide correspond to residues 967–991 of INF2.

### The H and B regions of the INF2 WH2/DAD are both required for actin binding

**Fig. 4:**
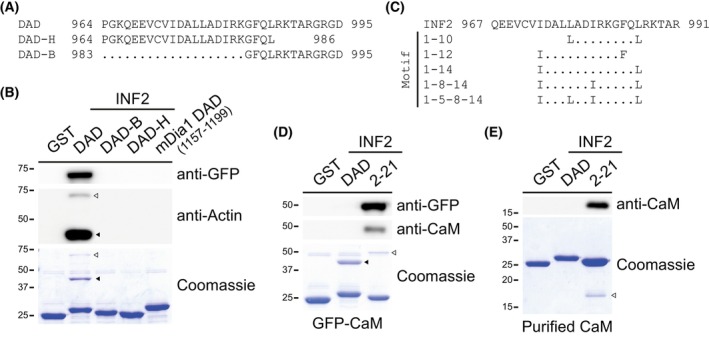
The hydrophobic and the basic regions of the WH2/DAD of INF2 are both essential for Actin binding. (A) Sequences of the INF2 WH2/DAD fragments used in the pull‐down analysis. (B) Pull‐down experiment using GST fused to the indicated WH2/DAD sequences of INF2 and DAD of mDia1 and extracts from GFP‐Actin‐expressing cells. The analysis was performed using antibodies against GFP and Actin. (C) Identification of different classes of consensus CaM‐binding site motifs in the INF2 WH2/DAD. (D) Pull‐down experiment using GST fused to the indicated INF2 sequences and extracts from GFP‐CaM‐expressing cells. Analysis was performed using antibodies against GFP and CaM. Purified GST fusions were stained with Coomassie blue to control for the amount of GST protein used (bottom panels in B and D). Note the presence of endogenous Actin in the GST‐DAD pull‐downs in both panels (B) and (D). (E) Pull‐down experiment using GST fused to the indicated INF2 sequences and purified CaM. Analysis was performed using antibodies against CaM. In the Coomassie blue‐stained gels, filled arrowheads indicate endogenous Actin (B, D), while empty arrowheads denote exogenous GFP‐Actin (B), GFP‐CaM (D), and purified CaM (E). Three independent experiments were performed in (B, D, E). A representative experiment is shown. CaM, calmodulin; DAD, diaphanous autoregulatory domain; GFP, green fluorescence protein; GST, glutathione S‐transferase.

To investigate how the deletion of the H or B region of the WH2/DAD of INF2 affects actin binding, we performed pull‐down experiments using purified glutathione S‐transferase (GST) fusion proteins made in *E. coli* and extracts of HEK293T cells expressing green fluorescence protein (GFP) fused to actin. The GST fusions included the INF2 964–995, which encompasses the entire WH2/DAD, as well as smaller fragments containing either the H or B region individually (Fig. 4A). Consistent with previous analyses using a larger INF2 fragment [17], the full WH2/DAD peptide was able to bind both exogenous GFP‐actin and endogenous actin. However, no actin binding was detected with the isolated H and B regions, nor with the WH2‐like DAD from mDia1, which lacks the conserved Leu residue in the LKKV box and served as a negative control (Fig. 4B). Therefore, the H and B regions are both required for actin to bind to the INF2 WH2/DAD.

The Calmodulin Target Database algorithm (http://calcium.uhnres.utoronto.ca/ctdb; [27]) predicts potential calmodulin‐binding sites (CaMBSs) based on structural and biophysical parameters such as hydrophobicity, hydrophobic moment, and propensity for α‐helix formation. Using this algorithm to search the entire INF2 sequence, we identified a high‐scoring CaMBS within the INF2 DAD‐H region. Furthermore, this region contains canonical CaMBS motifs (Fig. 4C), making it a strong candidate to bind calmodulin (CaM). To test whether the INF2 DAD‐H region functions as a CaMBS, we used this region fused to GST in pull‐down assays with extracts of GFP‐CaM‐expressing cells. As a positive control, we used the 2–21 peptide of the INF2 N‐terminal extension, previously shown to bind CaM [28]. While GFP‐CaM successfully bound to the 2–21 peptide, no binding was observed with the INF2 WH2/DAD, indicating that the H region of the WH2/DAD of INF2 does not function as a CaMBS (Fig. 4D). Consistent with the results shown in Fig. 4B, binding of endogenous actin was detected with the INF2 fragment encompassing the entire WH2/DAD (Fig. 4D, bottom panel). This raised the possibility that actin might compete with CaM for binding to the INF2 WH2/DAD. To rule out this possibility, we performed pull‐down assays using purified CaM. We observed no binding to the WH2/DAD, indicating that actin and CaM do not compete for WH2/DAD binding (Fig. 4E).

### Effect of WH2/DAD deletions on INF2 activity

**Fig. 5:**
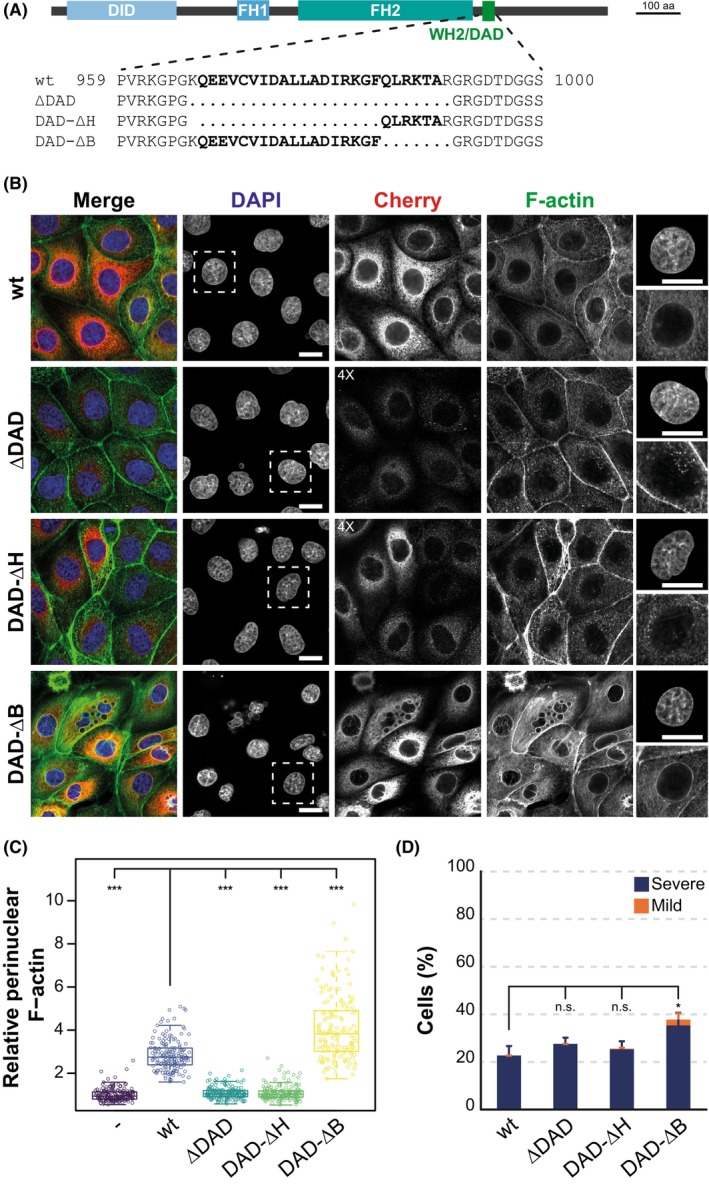
The H region, but not the B region, of the WH2/DAD is essential for Actin polymerization by INF2. (A) Schematic representation of the INF2 deletion mutants expressed in this study. (B–D) INF2 KO MDCK cells expressing, or not expressing, Cherry fusions with the indicated INF2 proteins, were stained to visualize F‐Actin. Nuclei were counterstained with DAPI. The contrast in the Cherry channel was increased as indicated to visualize the cells expressing INF2 ΔDAD and INF2 DAD‐ΔH. An enlarged view of the perinuclear region (boxed) from representative cells of each condition is shown in the rightmost panels, displaying DAPI (top) and F‐Actin (bottom) fluorescence images. Scale bars, 15 μm (B). (C) The graph shows the relative intensity of F‐Actin staining in the perinuclear region compared with control INF2 KO cells, measured across three equatorial planes. Statistical significance was assessed using the Mann–Whitney–Wilcoxon test. (D) Percentage of INF2 KO cells with mild (single micronucleus, a multilobed nucleus, or two normal nuclei) or severe (multiple micronuclei) nuclear phenotypes. Statistical significance was determined using a two‐tailed, unpaired Student's *t*‐test. > 150 cells (C) and > 300 cells (D) were analyzed for each experimental condition; three independent experiments; ns, not significant; *P < 0.01; ***P < 0.001. DAD, diaphanous autoregulatory domain; DAPI, 4′,6‐diamidino‐2‐phenylindole dihydrochloride; DID, diaphanous inhibitory domain; FH, formin homology; WH2, WASP homology 2; wt, wild type.

To study the functional impact of WH2/DAD deletions in INF2, we used INF2 knockout (KO) MDCK cells. This cell model was selected to eliminate potential artifacts from interactions with endogenous INF2 and to facilitate the detection of variation in F‐actin levels, since these cells exhibit low basal levels of F‐actin [7, 28]. INF2 exists as two isoforms: INF2‐1 (also known as INF2‐CAAX), which localizes to the endoplasmic reticulum, and INF2‐2 (INF2‐non‐CAAX), which is cytosolic [29, 30]. We focused on INF2‐1 (referred to as INF2 hereafter) because both isoforms function similarly when expressed exogenously [7]. We expressed Cherry alone or Cherry‐fused INF2 constructs with various WH2/DAD deletions: INF2 with the entire WH2/DAD deleted (INF2 ΔDAD), INF2 with the WH2/DAD‐H region deleted (INF2 DAD‐ΔH), and INF2 with the WH2/DAD‐B region deleted (INF2 DAD‐ΔB) (Fig. 5A). We then compared their effect on perinuclear F‐actin levels to those of wild‐type INF2 (Fig. 5B,C). Notably, both wild‐type INF2 and INF2 DAD‐ΔB increased perinuclear F‐actin levels in INF2 KO cells, with the effect being more pronounced in cells expressing INF2 DAD‐ΔB. In contrast, neither INF2 ΔDAD nor INF2 DAD‐ΔH had a significant impact on F‐actin levels. Consistent with previous findings that INF2 proteins with deregulated activity induce nuclear aberrations [7], we observed that INF2 DAD‐ΔB, but not INF2 ΔDAD or INF2 DAD‐ΔH, produced such detrimental effects (Fig. 5D).

In summary, INF2 requires the WH2/DAD‐H region, but not the WH2/DAD‐B region, region for actin polymerization.

### Analysis of the expression of natural variants in the INF2 WH2/DAD

**Fig. 6:**
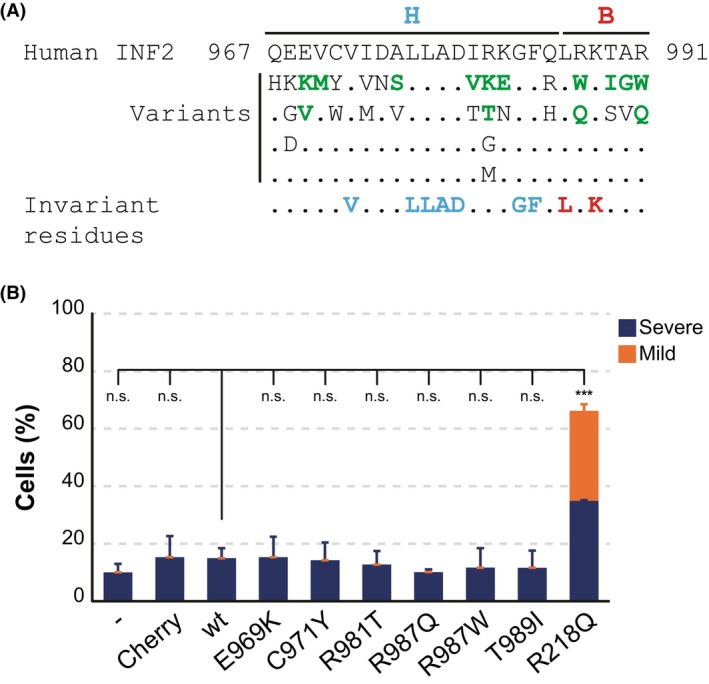
Expression of natural INF2 WH2/DAD variants does not affect nuclear morphology. (A) Compilation of allelic variants in the WH2/DAD of INF2 found in the general human population. Variants annotated in the ClinVar database are highlighted in green (See Table S1). Invariant residues are highlighted in blue (hydrophobic region, H) and red (basic region, B). (B) Percentage of MDCK cells expressing the indicated INF2 WH2/DAD variants displaying mild (single micronucleus, a multilobed nucleus, or two normal nuclei) or severe (multiple micronuclei) nuclear phenotypes. The pathogenic INF2 R218Q DID variant was included as a control. Statistical significance was determined using a two‐tailed, unpaired Student's *t*‐test. > 300 cells were analyzed for each experimental condition; three independent experiments; n.s., not significant; ***, *P* < 0.001. wt, wild type.

Although mutations in the INF2 WH2/DAD have not been genetically linked to human disease, the DAD domains play a crucial role in regulating formin activity and, potentially, mutations in the WH2/DAD could contribute to disease. Thirty‐two variants in the INF2 WH2/DAD were found in public human databases (Fig. 6A). An earlier study established a strong correlation between the pathogenicity of INF2 DID variants and the formation of nuclear morphological aberrations in MDCK cells [7]. Specifically, only the disease‐linked variants, not the benign ones, caused abnormalities. To investigate whether the expression of INF2 WH2/DAD variants affects nuclear morphology, we expressed six variants (E969K, C971Y, R981T, R987W, R987Q, T989I) classified as probably being deleterious (Table S1) by four algorithms (AlphaMissense, PROVEAN, SIFT, and Polyphen‐2) that predict the possible impact of mutations from complementary perspectives. While the expression of the pathogenic INF2 R218Q DID variant produced a dramatic nuclear phenotype with multi‐micronucleated cells and other nuclear defects, the WH2/DAD mutants, as well as control INF2, did not induce significant alterations in nuclear morphology (Fig. 6B). Thus, in this assay, the WH2/DAD mutants behaved similarly to the benign group of DID variants [7].

## Discussion

The DAD of formins in the mDia family plays a dual role: it mediates autoinhibition by interacting with the DID and facilitates actin nucleation [14]. Sequences similar to the mDia DAD are present in formins throughout the eukaryotes, although not all formins possess a DAD, nor are all DAD‐containing formins regulated by autoinhibition [1, 9]. In our study, we have analyzed the WH2/DAD of INF2 and found its sequence to be highly conserved in vertebrates. We observed that its hydrophobic and basic regions form a single α‐helix in 30% TFE. Deletion of the entire WH2/DAD or solely the H segment rendered INF2 inactive, while removal of the B region led to an increase in cellular F‐actin levels and, as seen with pathogenic INF2 DID variants, caused nuclear aberrations. However, none of the six WH2/DAD natural variants we selected from the variants annotated in human public databases altered nuclear morphology when tested. This suggests that although it is theoretically possible, INF2 WH2/DAD variants with deleterious effects in cultured cells are either extremely rare or do not occur in humans.

The H region of WH2 domains and the H region of the mDia1 DAD adopts an α‐helical configuration in WASP WH2‐actin and mDia1 DAD‐DID crystals, respectively. In WH2 domains, the H region folds into an N‐terminal α‐helix that binds the mostly hydrophobic target‐binding cleft at the barbed end of the actin monomer, while the B segment extends along the actin surface as an elongated structure, roughly following the ridge between the outer and inner domains of the actin monomer [25, 26]. In mDia1 DAD, the H region forms an amphipathic α‐helix that establishes extensive hydrophobic contacts with a shallow groove in the DID domain before undergoing a 90° bend. Beyond this point, the remainder of the DAD, including the B region, is not visible in the crystal structure. It has been suggested that this segment instead continues as part of an α‐helix [11, 12, 23, 24]. However, in the isolated INF2 WH2/DAD fragment, we observed no such bend between the H and B regions in either aqueous solution or 30% TFE. This suggests that if this conformation represents the structure of the WH2/DAD in the open form of INF2, then the B region must undergo a conformational change to facilitate binding to actin.

*In vitro* studies have shown that the roles of the mDia1 WH2‐like/DAD in nucleation and autoinhibition can be functionally separated. Mutations in the B region severely impair nucleation activity, whereas specific mutations in the H region impair autoinhibition, with minimal or no effect on nucleation [14, 31, 32]. Although the B region is not necessary for H region binding to the mDia DID, its presence significantly strengthens the binding affinity relative to the H region alone [11, 12, 23]. This increase in binding affinity is also observed in the WH2 domain‐containing WIP and WAVE proteins [26]. Our results are consistent with this, as the expression of INF2 DAD‐ΔB, but not of INF2 ΔDAD or INF2 DAD‐ΔH, led to increased F‐actin levels, indicating that the H region is sufficient for actin polymerization in INF2. Although our pull‐down assays demonstrated that the full WH2/DAD peptide binds actin, no binding was observed when the H and B regions were tested separately, suggesting that while the H region contributes to actin nucleation, the presence of both regions optimizes binding efficiency. This is similar to what occurs in the WH2‐like DAD of mDia1, which binds actin with more than 1000 times lower affinity than the INF2 WH2/DAD [33], and like the INF2 WH2/DAD‐H region, failed to bind actin at detectable levels in our pull‐down assays.

In mDia1, the DAD interacts with the DID, maintaining the protein in an inactive, closed conformation. However, in INF2, the DID‐DAD interaction is approximately 70 times weaker than in mDia1, making this affinity insufficient for effective autoinhibition. A complex formed by cyclase‐associated protein (CAP) and lysine‐acetylated actin (KAc‐actin) enhances this interaction approximately five‐fold, acting as a bridge between the DID and the WH2/DAD [13]. CAP binds to the N‐terminal segment of INF2 spanning residues 1–420 (which contains the DID), while KAc‐actin interacts with the segment spanning residues 941–1249, probably binding to the WH2/DAD [34]. As mentioned earlier, deleting the entire WH2/DAD renders INF2 inactive, likely due to loss of nucleation activity. Notably, INF2 DAD‐ΔB showed higher polymerization activity than wild‐type INF2, suggesting that the B region plays a regulatory role in modulating INF2 activity. Consistent with previously reported correlations between actin levels and nuclear aberrations by the expression of deregulated, pathogenic forms of INF2 [7], the expression of INF2 DAD‐ΔB, but not of INF2 ΔDAD or INF2 DAD‐ΔH, induced nuclear morphological changes. However, the nuclear alterations caused by INF2 DAD‐ΔB were milder than those caused by the pathogenic INF2 R218Q DID variant, probably due to residual regulation of INF2 activity.

*In vitro* experiments with purified INF2 fragments have shown that, in addition to promoting actin polymerization, INF2 can sever actin filaments. This activity is not observed in other formins. An INF2 fragment containing the FH1‐FH2‐WH2/DAD domains, but not the FH1‐FH2 fragment alone, is capable of severing actin filaments *in vitro*. This severing activity is lost when three critical leucine residues in the WH2 are mutated [17]. As a further evidence of this INF2 activity, structural studies show that the FH2 and WH2/DAD domains of INF2 are appropriately positioned to sever actin filaments, a configuration absent in mDia1, which is not suited for severing [19]. It is difficult to detect filament severing in cultured cells because of the degree of disruption required for this activity to become noticeable, hindering the confirmation of this activity within a cellular environment. If INF2 severs actin filaments in cells as it does *in vitro*, it is likely that the H and B regions are both required and would be defective in our INF2 WH2/DAD deletion mutants.

In addition to INF2, human DAAM1 and DAAM2 contain sequences (LKRN and LKRS, respectively) in the DAD‐B region that meet the criteria for the LKKV box of WH2 domains. The latter residue in this tetrapeptide varies across different WH2 domains. For example, a serine occupies this position in the first WH2 domain of Spire and Vopl, and in the second WH2 domain of N‐WASP [16]. Therefore, it is possible that the DAD of DAAM1 and DAAM2 also functions as a WH2 domain.

Formins of the mDia subfamily, as well as other DID‐ and DAD‐containing formins, are activated by the binding of Rho GTPases in their GTP‐loaded active form to the DID and the adjacent N‐terminal region, which disrupts the autoinhibitory DID–DAD interaction [1]. In contrast, INF2 activation is mediated by the binding of Ca^2+^/CaM [35]. Ca^2+^/CaM binds to the first α‐helix of a 35‐amino acid extension N‐terminal to the DID. Mutation of this site completely abolishes CaM binding to full‐length INF2, suggesting that this is the only CaMBS of INF2 [28]. However, this does not rule out the possible presence of cryptic CaMBSs that may become accessible upon activation. Although algorithms predict the presence of potential CaMBSs within the INF2 WH2/DAD and identify several consensus CaMBS motifs, our experiments showed that this region does not bind Ca^2+^/CaM.

The comparison of the INF2 WH2/DAD across vertebrates and within the human population revealed that, in addition to the three Leu residues (Leu976, Leu977, and Leu986) that are essential for WH2 function [21], some residues are highly conserved. Notably, no substitutions were found in the Asp979 and the Gly‐Phe dipeptide (residues 983 and 984) of the H region, or the Lys988 within the LKRT sequence of the B region. Only strict conservative replacements were observed for Val972 and Arg981, which were substituted by Ile and Lys, respectively. This extremely rigid conservation strongly suggests that these residues are critical for the correct function of the INF2 WH2/DAD. It is significant that none of the six natural missense variants selected for their high pathogenic potential from the INF2 WH2/DAD variants found in the human population induced nuclear aberrations in cultured cells, behaving similarly to the benign group of INF2 DID variants. Given the previously established correlation between nuclear aberration in cultured cells and disease‐causing INF2 variants in humans, we conclude that the WH2/DAD mutations annotated in public databases are unlikely to cause focal segmental glomerulosclerosis or Charcot–Marie–Tooth disease. Notably, five (E969K, R981T, R987Q, R987W, T989I) of the six WH2/DAD variants tested, along with nine additional variants, are documented in the ClinVar database, which tracks disease associations and clinical involvement. These fourteen variants have been found in individuals with renal and neurological conditions, among others (Table S1). Therefore, it is plausible that some of the INF2 WH2/DAD variants might not be pathogenic on their own but could require a second genetic hit to contribute to disease development.

In conclusion, our study draws attention to the unique structural and functional characteristics of the INF2 WH2/DAD, highlighting the crucial role of the H region in actin polymerization and the potential regulatory significance of the B region. These findings enhance our understanding of INF2's distinctive activity, providing new insights into its cellular functions in actin dynamics and its involvement in disease mechanisms.

## Materials and methods

### Cells and cell culture

Epithelial canine MDCK II (CVCL_0424) INF2 KO cells [36] and human epithelial HEK293T (CVCL_0063) cells from the American Type Culture Collection (https://www.atcc.org) were grown in minimal essential medium and Dulbecco's modified Eagle's medium, respectively, each supplemented with 5–10% (v/v) fetal bovine serum. Cells were cultured at 37 °C in a humidified incubator with 95% air/5% CO_2_ atmosphere. Regular mycoplasma testing was conducted to ensure cell‐line integrity.

### DNA constructs

INF2‐1 proteins fused to the C terminus of mCherry were expressed using the retroviral p33Cherry vector [28]. Point mutations in INF2‐1 were introduced with the Quick‐Change II directed mutagenesis kit using primers with the desired modifications. Internal deletions in INF2 (INF2‐ΔDAD, INF2‐DAD‐ΔH, and INF2‐DAD‐ΔB) were generated by site‐directed mutagenesis by overlap extension PCR using wild‐type INF2‐1 as the template and specific oligonucleotides [37]. GST DNA constructs were generated in the pGEX‐4EX vector by cloning annealed synthetic oligonucleotides corresponding to the indicated DAD sequences of INF2 (GST‐DAD, GST‐DAD‐H, GST‐DAD‐B) or mDia1 (mDia1 DAD) between the BspE I and Xho I sites. All constructs were verified by DNA sequencing (Macrogen Spain, Madrid, Spain).

### Retroviral infections

To generate retroviral particles, HEK293T cells grown in p100 culture dishes were cotransfected with p33Cherry constructs encoding the indicated mCherry‐INF2 fusion proteins, along with the helper plasmids MLV‐GagPol/pHIV 8.1 and pHIT VSVg (1.4 μg·mL^−1^) in a 4.6 : 3.3 : 1 ratio using polyethyleneimine [38]. After 48 h, the supernatant containing viral particles was collected and filtered and used to infect MDCK cells. Cells were treated with 10 μg·mL^−1^ polybrene at 37 °C for 15 min before exposure to the viral supernatant for 7 h. Cells were subsequently washed and incubated for 48 h before further analysis.

### Confocal microscopy

Cells were fixed with 10% formalin (derived from 37% formaldehyde solution) and permeabilized using 0.2% Triton X‐100 for 5 min on ice. Samples were treated with 10 mm glycine in phosphate‐buffered saline for 5 min to quench the aldehyde groups. After blocking with 3% (wt/v) bovine serum albumin for 30 min, cells were stained with Alexa 488‐conjugated phalloidin and 4′,6‐diamino‐2‐phenylindole dihydrochloride (DAPI). To facilitate visualization of transfected cells, cells were also stained with anti‐Cherry antibodies followed by anti‐rabbit IgG antibodies conjugated to Alexa 555. After extensive washing, coverslips were mounted on glass slides with Fluoromount. Fluorescence was examined with a Nikon A1R+ confocal laser‐scanning microscope equipped with a 60× water immersion objective (NA 1.2). Brightness and contrast were optimized with Fiji software (https://fiji.sc), and laser‐scanning microscopy images were converted to TIFF format. The levels of perinuclear F‐actin were quantified by measuring fluorescence in a 0.5‐μm‐wide region of interest surrounding the nucleus. Data represent the average intensity from three focal planes. imagej (https://imagej.nih.gov/ij/) was used for image quantification and brightness and contrast optimization.

### GST pull‐down assay

GST‐fusion proteins were expressed in *E. coli* BL21 cells. Cells were grown in LB media at 37 °C until reaching an OD_600_ of 0.6–0.8. Protein expression in bacteria was induced with 0.5 mm isopropyl‐β‐*D*‐1‐thiogalactopyranoside at 20 °C for 16 h. HEK293T cell lysates expressing GFP‐actin or GFP‐CaM were prepared in 20 mm Tris/HCl, pH 8.0, 150 mm NaCl, 1% NP40, 1.15% glycerol, 1 mm sodium orthovanadate, 0.1 mm PMSF, 2.5 mm CaCl_2_, and a commercial protease inhibitor cocktail. After centrifugation at 14 000 rpm in a microcentrifuge for 5 min to remove nuclei, the cell extracts were incubated with 30 μg of the indicated GST‐fused proteins immobilized on glutathione‐Sepharose beads at 4 °C. For the pull‐down experiment with purified CaM (Merck Life Science S.L.U., Madrid, Spain), the immobilized GST‐fused proteins were incubated with 2 μg of purified CaM. After 3 h incubation, beads were pelleted by centrifugation at 800 rpm for 5 min and washed three times with cold PBS. Samples were analyzed by immunoblotting. Aliquots from bead‐bound GST‐fusion proteins were stained with Coomassie blue to control for the amount of protein used.

### Immunoblotting

Membranes were blocked with 5% BSA (w/v) and 0.05% (v/v) Tween‐20 in Tris‐buffered saline before overnight incubation with the appropriate primary antibodies. Following washes with Tris‐buffered saline containing 0.05% Tween 20, membranes were incubated for 30 min with horseradish peroxidase‐conjugated secondary antibodies. Signals were visualized using Clarity Western ECL substrate (Bio‐Rad, Madrid, Spain).

### Synthetic peptide

The WH2/DAD peptide of human INF2 (residues 967–991) was synthesized with a Cys971Ser substitution to prevent potential instability due to oxidation and disulfide bond formation. The peptide's N and C termini were protected by acetylation and amidation, respectively. It was synthesized using standard Fmoc [N‐(9‐fluorenyl)methoxycarbonyl] solid‐phase methods and purified via reverse‐phase high‐performance liquid chromatography (CASLO ApS, Kongens Lyngbay, Denmark). The final peptide had a purity of 98.94%, with a reverse‐phase‐HPLC: retention time of 15.56 min (linear 28–50% B gradient over 22 min; buffer A: 0.05% TFA in H_2_O/CH_3_CN 98 : 2; buffer B: 0.05% TFA in H_2_O/CH_3_CN 1 : 9). Liquid chromatography‐high resolution mass spectrometry confirmed a molecular weight of 2900.14 for the peptide, consistent with the calculated value of [M + H]^+^ of 2898.38.

### Circular dichroism

CD spectra were recorded on a Jasco J‐810 spectropolarimeter equipped with a Peltier temperature control unit. INF2 WH2/DAD peptide stock solution was prepared at 1 mg·mL^−1^ in milliQ‐water. The peptide concentration was 50 μm in both aqueous and mixed solvent (30% TFE/70% H_2_0) at pH 5.5. Spectra were recorded at 25 °C in a 1‐mm path‐length quartz glass cell (Suprasil, Hellma, Müllheim, Germany) from 260 to 190 nm at 0.1 nm intervals. Isothermal spectra were obtained at a scan speed of 50 nm·min^−1^, a response time of 4 s, and a bandwidth of 1 nm. Baseline corrections were applied, and the resulting spectra were averaged over four scans. Data were processed using the adaptive smoothing method available in the Jasco Spectra Analysis software and reported in molar ellipticity units ([*θ*], deg·cm^2^·dmol^−1^). Helix content estimations were based on the [*θ*] values at 222 nm ([*θ*]^222nm^, deg·cm^2^·dmol^−1^), applying a standard formula.

### NMR spectroscopy

The NMR methods used in this study have been described in detail previously [28]. NMR spectra were acquired using a Bruker Avance‐600 spectrometer operating at a proton frequency of 600.13 MHz, equipped with a cryoprobe. Samples were prepared by dissolving the lyophilized peptide in aqueous solution (H_2_O/D_2_O 9 : 1 v/v or D_2_O) or 30% TFE (30% [D3]‐TFE/70% H_2_O/D_2_O 9 : 1 v/v or 30% [D3]‐TFE/70% D_2_O) at 0.5–1.0 mm. Sodium 2,2‐dimethyl‐2‐silapentane‐5‐sulfonate (0.1 mm) was added as an internal reference for ^1^H chemical shifts. If required, pH was adjusted to 5.5 with minimal amounts of NaOD or DCl. 1D and 2D spectra, including ^1^H,^1^H double‐quantum filtered correlation spectroscopy, ^1^H,^1^H total correlation spectroscopy (TOCSY), ^1^H,^1^H nuclear Overhauser effect spectroscopy (NOESY), and ^1^H‐^13^C heteronuclear single quantum coherence (HSQC) spectra at ^13^C natural abundance, were acquired using standard pulse sequences, with water suppression using an excitation sculpting scheme. Spectra were processed using Topspin 4.0.8 (https://www.bruker.com/en/products‐and‐solutions/mr/nmr‐software). Baseline correction was applied in both dimensions. ^13^C *δ*‐values were indirectly referenced using the IUPAC‐IUB recommended ^1^H/^13^C chemical shift ratio (0.25144953) [39]. Chemical shifts were assigned with NMRFAM‐SPARKY (https://nmrfam.wisc.edu/nmrfam‐sparky‐distribution), and ^1^H and ^13^C chemical shift values were deposited in BioMagResBank (https://bmrb.io) with accession codes 52961 for aqueous solution and 52941 for TFE solution. Δ*δ*_Hα_ values were obtained from the equation: Δ*δ*_Hα_ = *δ*_Hα_^observed^ – *δ*_Hα_^RC^, ppm, where *δ*_Hα_^observed^ is the observed chemical shifts for ^1^Hα nuclei, and *δ*_Hα_^RC^ is the reference random coil values for the ^1^Hα chemical shifts. These reference values were taken from Wishart *et al*. [40]. The random coil ranges are −0.04 ppm < Δ*δ*_Hα_ < +0.04 ppm. As previously reported [41], assuming a two‐state folded/unfolded transition, the helix percentage was obtained by multiplying by 100 the value resulting from the division of the Δ*δ*_Hα_ values, averaged for the helical residues, by 0.39 ppm, which is the mean Δ*δ*_Hα_ at protein α‐helices. Assuming an experimental error of ±0.01 ppm in the measurement of ^1^H *δ*‐values, the errors in the estimated populations were ±3%.

### Peptide structure calculation

Peptide structure was determined using the standard iterative protocol for automatic NOE assignment of the cyana 3.98 program (http://www.bpc.uni‐frankfurt.de/guentert/wiki/index.php/Software). Seven cycles of NOE assignment and structure calculation were run, with 100 conformers calculated per cycle. Input data included (1) assigned chemical shifts, (2) NOE integrated cross‐peaks from 150 ms NOESY spectra, and (3) *φ* and *ψ* dihedral angle restraints. NOE cross‐peaks were integrated using the automatic integration subroutine of nmrfam‐sparky software (https://nmrfam.wisc.edu/nmrfam‐sparky‐distribution). Restraints for dihedral angles were obtained from ^1^H and ^13^C chemical shifts using TALOS‐N (https://spin.niddk.nih.gov/bax/nmrserver/talosn). The final structure, which is the ensemble of the 20 lowest target function conformers calculated in the final cycle, was visualized with MOLMOL (https://sourceforge.net/p/molmol). The structural ensembles obtained in aqueous solution and in 30% TFE have been deposited in the Protein Data Bank under accession codes 9RWF and 9G7T, respectively.

### Bioinformatic analyses

NCBI blast (https://blast.ncbi.nlm.nih.gov) was employed to align the human INF2 WH2/DAD sequence with those from 45 vertebrates and with the DAD sequence of other humans' formins. INF2 WH2/DAD variants in the human population were searched using public databases including dbSNP (ncbi.nlm.nih.gov/snp), gnomAD (gnomad.broadinstitute.org), LOVD3 (lovd.nl), TOPMED (topmed.nhlbi.nih.gov), and ClinVar (ncbi.nlm.nih.gov/clinvar).

### Statistical analysis

Graphs and statistical analyses were performed using RStudio (v1.2.5033; https://www.rstudio.com) and microsoft excel (https://www.microsoft.com/en‐us). Differences between means were assessed using two‐tailed, unpaired Student's *t*‐tests, and median differences were evaluated with the Mann–Whitney‐Wilcoxon test, as indicated. Additional information is provided in the figure legends.

### Materials and software

The materials and software used, along with their sources, are listed in Table S2.

## Conflict of interest

The authors declare no conflict of interest.

## Author contributions

LL‐H and LF‐M performed and analyzed the pull‐down experiments and the cell‐based experiments under the supervision of IC; PM and MAJ conducted the NMR analyses; LL‐H carried out the bioinformatic analyses and prepared the figures; IC and MAJ contributed to data interpretation, writing, and critically reviewing the final version; MAA designed the study, supervised the work, and wrote the manuscript.

## Supporting information


**Fig. S1.** Conservation of the WH2/DAD of INF2 across vertebrates.
**Table S1.** Pathogenicity predictions and disease association of INF2 Cys971Tyr and the fourteen INF2 variants annotated in ClinVar.
**Table S2.** Materials and software.

## Data Availability

The datasets generated during and/or analyzed during the current study are available from the corresponding author upon request. ^1^H and ^13^C chemical shift values have been deposited in the BioMagResBank under accession codes 52961 (aqueous solution) and 52941 (30% TFE solution). The structural ensembles corresponding to the INF2 WH2/DAD peptide in H_2_O and in 30% TFE have been deposited in the Protein Data Bank under accession codes 9RWF and 9G7T, respectively.
