# Supplementary material for: Structural and functional dissection of the WH2/DAD motif of INF2, a formin linked to human inherited degenerative disorders
Source: FEBS J. 2025 Sep 24;293(3):825–41. doi: 10.1111/febs.70271 (PMC12871907; doi:10.1111/febs.70271)
Supplement: Supplementary file 1 — Fig. S1. Conservation of the WH2/DAD of INF2 across vertebrates. Table S1. Pathogenicity predictions and disease association of INF2 Cys971Tyr and the fourteen INF2 variants annotated in ClinVar. Table S2. Materials and software. [file FEBS-293-825-s001.pdf]

Species	Accession number	Peptide		
**Homo sapiens**	**Q27J81.2**	**967**	**Q-EEVCVIDALLADIRKGFQLRKTAR**	**991**
Pongo abelii	PNJ26083.1	923	.-........................	947
Pan troglodytes	XP_016782299.1	997	.-........................	1021
Macaca mulatta	XP_028707767.1	979	.-........................	1003
Canis lupus dingo	XP_025298787.2	973	.-........................	997
Ursus arctos horribilis	XP_026370957.1	970	.-........................	994
Felis catus	XP_023111892.1	972	.-.D......................	996
Acinonyx jubatus	XP_026922562.1	996	.-.D......................	1020
Lontra canadensis	XP_032695402.1	956	.-........................	980
Molossus molossus	KAF6500422.1	984	.- ....R......	1008
Equus caballus	XP_023484361.1	962	.-........................	986
Callithrix jacchus	XP_035117579.1	998	.-........................	1022
Camelus ferus	XP_032338697.1	980	.-........................	1004
Trichechus manatus latirostris	XP_004376812.1	974	.- ....V.	998
Microtus ochrogaster	XP_026637264.1	1005	.-........................	1029
Mus musculus	ABI20145.1	1000	.-........................	1024
Phoca vitulina	XP_032261917.1	983	.-........................	1007
Suricata suricatta	XP_029807100.1	1025	.-........................	1049
Sarcophilus harrisii	XP_031809000.1	981	.-.......................K	1005
Ornithorhynchus anatinus	XP_028920082.1	995	.P.......................K	1019
Meleagris gallopavo	XP_010710280.1	955	.-.D...............T....KN	979
Gallus gallus	XP_004936455.1	1036	.-.D...............T....KN	1060
Struthio camelus australis	KFV81645.1	904	.-.D...............T....—-	926
Oxyura jamaicensis	XP_035182672.1	1022	.-.................T....KN	1046
Chiroxiphia lanceolata	XP_032547290.1	1014	.-D................T....KN	1038
Apteryx rowi	XP_025931741.1	988	.-.D...............T....KN	1012
Parus major	XP_015485972.1	989	.-.................T....KN	1013
Calypte anna	XP_030307864.1	998	.-.................A....KN	1022
Cyanistes caeruleus	XP_023783218.1	986	.-.................T....KN	1010
Zootoca vivipara	XP_034960855.1	989	.-D.......................	1013
Podarcis muralis	XP_028605128.1	1013	.-D.......................	1037
Pogona vitticeps	XP_020633002.1	981	.-....I...................	1005
Python bivittatus	XP_025019713.1	960	.-......................N.	984
Protobothrops mucrosquamatus	XP_015670109.1	1023	.-......................I.	1047
Chelonia mydas	EMP28055.1	736	.-D............K.........K	760
Trachemys scripta elegans	XP_034623756.1	966	.-......................VK	990
Xenopus laevis	NP_001084562.1	1030	L-..G.I..D.....K.........K	1054
Xenopus tropicalis	NP_001072591.1	1030	L-..G.I........K.........K	1054
Bufo bufo	XP_040268406.1	1003	I-..G.I........K.........K	1027
Geotrypetes seraphini	XP_033807612.1	1091	L-..G.I........K.........K	1115
Oryzias latipes	XP_011488755.1	897	.-NDG.I..H.........S....RP	921
Echeneis naucrates	XP_029349421.1	973	P-NDG.I..H.........S....RP	997
Hippoglossus stenolepis	XP_035023511.1	912	H-DDG.I..H.........S...PRP	936
Oncorhynchus tshawytscha	XP_024293225.1	903	.-DDG.I..H..N......H....RS	927
Amblyraja radiata	XP_032882930.1	991	.-..E.I..V..S......K.K.AT.	1015

Mammals **Birds** **Reptiles** **Amphibians** **Fishes**

Figure S1. **Conservation of the WH2/DAD of INF2 across vertebrates**. Alignment of the sequence of the 967-991 WH2/DAD sequence of human INF2 with the corresponding sequence from 45 species representing various vertebrate groups. The alignment was performed with the BLAST program. WH2/DAD, WASP homology 2/diaphanous autoregulatory domain.

Table S1. **Pathogenicity predictions and disease association of INF2 Cys971Tyr and the fourteen INF2 DAD variants annotated in ClinVar**

**Protein variant**	**AlphaMissense**	**PROVEAN**	**SIFT**	**PolyPhen-2**	**ClinVar**
**p.Glu969Lys**	Ambiguous	Deleterious	Damaging	Possibly damaging	FSGS+CMT
p.Glu969Val	Likely benign	Deleterious	Damaging	Possibly damaging	FSGS+CMT
p.Val970Met	Likely benign	Neutral	Damaging	Probably damaging	FSGS+CMT/FSGS
**p.Cys971Tyr**	Likely pathogenic	Deleterious	Damaging	Probably damaging	---
p.Ala975Ser	Likely benign	Neutral	Tolerated	Possibly damaging	FSGS+CMT
p.Ile980Val	Likely pathogenic	Neutral	Damaging	Possibly damaging	FSGS+CMT/ Inborn genetic diseases
p.Arg981Lys	Likely benign	Neutral	Tolerated	Possibly damaging	FSGS+CMT
**p.Arg981Thr**	Likely pathogenic	Deleterious	Damaging	Probably damaging	FSGS+CMT
p.Lys982Glu	Likely pathogenic	Deleterious	Tolerated	Probably damaging	FSGS+CMT
**p.Arg987Trp**	Likely pathogenic	Deleterious	Damaging	Possibly damaging	FSGS+CMT
**p.Arg987Gln**	Ambiguous	Deleterious	Damaging	Probably damaging	FSGS+CMT
**p.Thr989Ile**	Likely pathogenic	Deleterious	Damaging	Probably damaging	Inborn genetic diseases
p.Ala990Gly	Likely benign	Neutral	Tolerated	Probably damaging	Inborn genetic diseases
p.Arg991Trp	Likely benign	Deleterious	Damaging	Probably damaging	FSGS+CMT
p.Arg991Gln	Likely benign	Neutral	Damaging	Benign	FSGS+CMT

The six variants tested in Fig. 6 are highlighted in bold. CMT, Charcot-Marie-Tooth disease; FSGS, focal segmental glomerulosclerosis.

Table S2. **Materials and software**

	SOURCE	IDENTIFIER
**Antibodies**		
Mouse mAb anti-calmodulin	Millipore	Cat#05-173
Mouse mAb anti-actin	Sigma	Cat#A4700
Rabbit polyclonal antibody anti-Cherry	Abcam	Cat#ab167453
Mouse mAb anti-GFP	Sigma-Aldrich	Cat#11814460001
Secondary donkey anti-mouse IgG antibody, Alexa Fluor 488-conjugated	Thermo Fisher	Cat#A-21202
Secondary donkey anti-rabbit IgG antibody, Alexa Fluor 555-conjugated	Thermo Fisher	Cat#A-21429
Secondary donkey anti-rabbit IgG antibody, HRP conjugated	GE Healthcare	Cat#NA934
Secondary donkey anti-mouse IgG antibody, HRP conjugated	Jackson Labs	Cat#715-035-151
**Bacterial strains**		
E. coli BL21	Stratagene	Cat#200131
E. coli XL10-Gold	Stratagene	Cat#200314
E. coli Rosetta 2(DE3)	Merck	Cat#71397
**Chemicals, peptide and proteins**		
Alexa Fluor™ 488 Phalloidin	Thermo Fisher	Cat#A12379
Calmodulin, bovine brain, high purity	Merck	Cat#208694
D_2O (99.9%)	Eurisotop	Cat#D216
[D_3]-2,2,2-trifluoroethanol (TFE), 99% pure	Eurisotop	Cat#D027BB
4’,6-diamidino-2-pheylindole (DAPI)	Merck	Cat#268298
Fetal bovine serum	Gibco	Cat#A4766801
Formalin solution, neutral buffered, 10%	Sigma-Aldrich	Cat#HT501128
Fluoromount	Merck	Cat#F4680
Glutathione Sepharose® 4B	Cytiva	Cat#GE17-0756-01
Isopropyl-β-D-1-thiogalactopyranoside (IPTG)	Apollo Scientific	Cat#BIMB 1008-106
Polybrene	Sigma-Aldrich	Cat#TR-1003-G
Polyethylenimine	Polysciences	Cat#23966
Protease inhibitor cocktail	Merck	Cat#11697498001
Sample buffer, Laemmli 2× concentrate	Sigma-Aldrich	Cat#S3401
Triton X-100	Merck	Cat#9036-19-5
Tween-20	Sigma-Aldrich	Cat#9005-64-5
Synthetic, modified INF2 WH2/DAD peptide (967-991)	CASLO ApS	N/A
**Commercial kits**		
Lipofectamine 2000	Thermo Scientific	Cat#11668019
QuickChange II Site-Directed Mutagenesis Kit	Stratagene	Cat#200523

Maxime PCR premix (iTaq)	LiliF Diagnostics	Cat#25026
T4 DNA ligase	Invitrogen	Cat#15224-017
Wizard® Plus SV Minipreps DNA Purification Systems	Promega	Cat#A1330
**Cell lines**		
MDCK II (CVCL_0424)	ATCC	Cat#CRL-2936
HEK293T (CVCL_0063)	ATCC	Cat#CRL-3216
**Recombinant DNA**		
pEGFP-hCaM	Addgene	Cat#47602
pEGFP-β-actin	This paper	N/A
p33Cherry	[28]	N/A
p33Cherry INF2-1	[28]	N/A
p33Cherry INF2-1 ΔDAD	This paper	N/A
p33Cherry INF2-1 DAD-ΔH	This paper	N/A
p33Cherry INF2-1 DAD-ΔB	This paper	N/A
p33Cherry INF2-1 Glu969Lys	This paper	N/A
p33Cherry INF2-1 Cys971Tyr	This paper	N/A
p33Cherry INF2-1 Arg981Thr	This paper	N/A
p33Cherry INF2-1 Arg987Trp	This paper	N/A
p33Cherry INF2-1 Arg987Gln	This paper	N/A
p33Cherry INF2-1 Thr989Ile	This paper	N/A
pGEX-4EX	[28]	N/A
pGEX-4EX INF2 (2-21)	[28]	
pGEX-4EX INF2 DAD (964-995)	This paper	N/A
pGEX-4EX INF2 DAD-H (964-986)	This paper	N/A
pGEX-4EX INF2 DAD-B (983-995)	This paper	N/A
pGEX-4EX mDia1 DAD (1157-1199)	This paper	N/A
MLV-GagPol/pHIV 8.1	[38]	N/A
pHIT VSVg	[38]	N/A
**Other**		
μ-Slide 8-well	Ibidi	80826
**Software**		
BLAST	https://blast.ncbi.nlm.nih.gov	
Topspin	https://www.bruker.com/en/products-and-solutions/mr/nmr-software	
NMRFAM-Sparky 3	https://nmrfam.wisc.edu/nmrfam-sparky-distribution	
TALOS-N	https://spin.niddk.nih.gov/bax/nmrserver/talosn	
Cyana 3.98	http://www.bpc.uni-frankfurt.de/guentert/wiki/index.php/Software	
MOLMOL	https://sourceforge.net/p/molmol	

ChimeraX	https://www.cgl.ucsf.edu/chimerax
Adobe Illustrator	https://www.adobe.com
Fiji	https://fiji.sc
Microsoft Excel	https://www.microsoft.com/en-us
R Studio	https://www.rstudio.com
